# Awareness, treatment, and practices of lifestyle modifications amongst diagnosed hypertensive patients attending the tertiary care hospital of Karachi: A cross-sectional study

**DOI:** 10.1016/j.amsu.2022.104587

**Published:** 2022-09-13

**Authors:** Sadia Yaqoob, Mahjabeen Yaseen, Furqan Ahmad Jarullah, Amna Saleem, Anmol Mohan, Mohammad Yasir Essar, Shoaib Ahmad

**Affiliations:** a Department of Medicine, Jinnah Medical and Dental College, Karachi, Pakistan; b Department of Nephrology, Fazaia Ruth Pfau Medical College, Karachi, Pakistan; c Department of Medicine, Karachi Medical & Dental College, Karachi, Pakistan; d Kabul University of Medical Sciences, Kabul, Afghanistan; e District Head Quarters Teaching Hospital, Faisalabad, Pakistan

**Keywords:** Hypertension, Awareness, Treatment, Practices, Control

## Abstract

**Background:**

Despite massive research guidelines, high blood pressure remains a major public health concern since barriers to treatment and control are on the rise. Lack of awareness is one of the serious impediments to managing hypertension. Therefore, this study is designed to gauge awareness, beliefs, and practices related to hypertension amongst diagnosed subjects.

**Methods:**

A total of 425 hypertensive patients were recruited from the wards and outpatient department of Jinnah Medical College Hospital located in Korangi district, Karachi. Data was obtained regarding socio-demographics, comorbidity, duration of HTN, current BP readings, and BMI. Additionally, awareness, practices, treatment, and control of hypertension were also assessed. Using IBM SPSS version 25.0, a chi-square test was run for categorical variables to analyze the differences in demographic variables, awareness, practices, and treatment between controlled and uncontrolled hypertensive patients. Multivariate regression model was used to identify the risk factors associated with uncontrolled hypertension. A p-value of less than 0.05 was considered significant.

**Results:**

65.6% of the total study population was females, of which, 70.7% had uncontrolled hypertension, with a p-value of 0.007. Majority of the candidates were between the ages of 50 and 59 and there was a significant difference in age groups of controlled and uncontrolled hypertensive patients (p-value = 0.019). Co-morbidities and duration of hypertension yielded no significant results. Awareness, treatment, and practices of lifestyle modifications amongst controlled or uncontrolled hypertension groups were not statistically significant. Age and female gender were the only risk factors significantly linked with uncontrolled hypertension.

**Conclusion:**

Overall, there was no significant difference in the statistics of controlled and uncontrolled hypertensive patients. This requires further investigation and evaluation to identify the unknown risk factors and co-morbid contributing to these findings. Most of the patients are on treatment and still not controlled, and this could be considered under treatment. Health professional's advice and counseling skills, social media, internet, and public awareness sessions can play an active role in the management of BP and its associated complications.

## Highlights

• Data regarding the awareness of lifestyle modifications in the general hypertensive population of Pakistan is scarce.
• Out of 425 hypertensive patients, 70.7% had uncontrolled hypertension. Age and female gender were the only risk factors significantly associated with uncontrolled hypertension.
• Most of the patients are on treatment and still not controlled, and thus could be considered under treatment. Interventions should be considered and implemented to potentially increase the control rates.

## 1 Introduction

Hypertension (HTN) is a chronic ailment that creeps up silently and quietly, giving off very few-if any-warning signs about the imminent destruction. Morbidity and mortality associated with disease progression continue to be an uphill struggle. Worldwide 1 billion of the population are stricken by hypertension and among them 7 million die per year due to inadequate control or complications secondary to uncontrolled Blood Pressure (BP) [1]. In light of this situation, costs of high blood pressure care have increased substantially. In 2021, the Centers for Disease Control and Prevention estimated the global economic burden of hypertension to be $131 to $198 billion per year [2].

By 2025 HTN prevalence is expected to expand up to 1.56 billion [3] and approximately two-thirds of the hypertensive patients happen to dwell in developing countries [4]. Leading causes of the burden of disease include genetic, metabolic, hormonal, and psychological factors like mental stress [5]. Other risk factors are namely smoking, low level of physical activity, high body mass index (BMI), excessive salt intake, alcohol consumption, hyperlipidemia, and diabetes mellitus [6].

Hypertension may lead to renal failure, cerebrovascular and cardiovascular events [7]. Globally complications of hypertension account for 9.4 million mortalities and 45% of all deaths are due to cardiovascular disease and 51% due to cerebrovascular disease [8]. The Eight Report of the Joint National Committee on Prevention, Detection, Evaluation, and Treatment of High Blood Pressure (JNC-8), WHO – ISH advises lifestyle modifications to all those who are at risk or have developed hypertension to prevent complications [9]. Recommended lifestyle changes include low salt intake, regular exercise or physical activity, adequate intake of fruits and vegetables, low-fat dairy products, cessation of smoking and moderation of alcohol intake [10].

Despite massive research guidelines, high blood pressure remains a major public health concern since barriers to treatment and control are on the rise. Lack of awareness is one of the serious impediments to managing hypertension. Unfortunately, local data regarding the awareness of lifestyle modifications in the general hypertensive population is scanty. Therefore, this study is designed to gauge awareness, beliefs, and practices related to hypertension amongst diagnosed subjects. This data can help us overcome the barrier at the patient level and provide better health care services to control BP and minimize the consequences of uncontrolled hypertension.

## 2 Methodology

This was an observational cross-sectional study, carried out after seeking the Ethical review board's approval between January and June 2019 (JMC.ERC.811.18), reported in line with the STROCSS criteria [11] and the Standards for Reporting Qualitative Research (SRQR) [12]. Using the following formula [13], n = Z [2] P(1−P)/d [2], where n = sample size, Z = 1.96 for the level of confidence of 95%, p = 0.462 (prevalence determined from a previous study [14]), and d is the precision set to 0.05, the sample size was estimated to be 382. A total of 425 patients were recruited from the wards and outpatient department of Jinnah Medical College Hospital located in Korangi district, Karachi. After informed verbal consent, a pretested structured questionnaire was interviewed face to face with known hypertensive patients. Self-reported physician diagnosis of hypertension, and SBP/DBP ≥140/90 sufficed the diagnosis of hypertension. Study participants over age 18 years and known cases of hypertension were included in this study while those with hypertension in association with dementia, psychoses, and schizophrenia were excluded.

The survey included socio-demographic data including age, gender, occupation, and education and other variables like co-morbidity, duration of HTN, current BP readings, and body mass index (BMI). The European health examination survey (EHES) guidelines were adopted to measure the blood pressure [15]. During the participant's visit to the hospital, three blood pressure measurements were obtained 1 min apart with an aneroid sphygmomanometer while the respondent was seated comfortably with the arm supported on a flat surface at heart level. The mean of three blood pressure readings was then used for analysis. Body Mass Index (BMI) was divided into four categories: underweight (<18.5), normal (18.5–24.5), overweight (25.0–29.9), and obese (≥30.00) [16].

Additionally, awareness, practices, treatment, and control of hypertension were also assessed. Concerning practices, individuals were evaluated for physical activity, smoking, and alcohol consumption, dietary intake of fruits and vegetables, and use of low-fat dairy products. According to the “International Physical Activity Questionnaire” [17], physical activity was categorized into low, moderate, and high. Hypertensive patients were labeled as controlled if the SBP/DBP were <140/90 mmHg with the use of anti-hypertensive agents, and <130/80 mmHg recommended for diabetic patients [18]. Uncontrolled hypertension was defined as SBP/DBP of ≥140/90 mmHg.

Data was entered and analyzed in IBM SPSS version 25.0 (IBM Corp, New York USA). N with percentages was computed for categorical variables while mean with standard deviation were observed for continuous data. A chi-square test was used for categorical variables to analyze the differences in demographic variables, awareness, practices, and treatment between controlled and uncontrolled hypertensive patients. A p-value of less than 0.05 was considered significant.

## 3 Results

In total, 425 people were included in the study. Females made up 279 (65.6%) of the total study population. Gender had a significant p-value of 0.007 when hypotheses were tested. Majority of the candidates were between the ages of 50 and 59, accounting for 144 (33.9%) of the population, with those aged 60 and older accounting for 131 (30.8%). There was a significant difference in age groups, and a p-value of 0.019 supported this finding.

168 (39.5%) of our sample size had no education, even at the primary level. Whereas 297 (69.9%) of our study population was unemployed at the time of the survey, 131 (44.1%) had reached retirement age. It was noted that 159 (37.4%) of the candidates were classified as overweight, while the remaining 147 (34.6%) were classified as obese. Family history revealed hypertension for 293 (68.9%) and diabetes mellitus for 198 (46.6%) participants. As promising as it appeared, the rest of the demographic data, including co-morbidities and duration of hypertension, yielded no significant results. Table 1 displays this demographic information.

**Table 1:** Comparison of demographics in patients with controlled and uncontrolled hypertension. *Statistically significant p-value (p < 0.05).

	Overall n (%) N = 425	Controlled hypertension n (%) N = 166	Uncontrolled hypertension n (%) N = 259	P-value
Age groups (years)				0.019*
20–29	10 (2.4)	5 (3.0)	5 (1.9)	
30–39	40 (9.4)	12 (7.2)	28 (10.8)	
40–49	100 (23.5)	27 (16.3)	73 (28.2)	
50–59	144 (33.9)	67 (40.4)	77 (29.7)	
60 or above	131 (30.8)	55 (33.1)	76 (29.3)	
Sex				0.007*
Male	146 (34.4)	70 (42.2)	76 (29.3)	
Female	279 (65.6)	96 (57.8)	183 (70.7)	
Level of education				0.622
No education	168 (39.5)	63 (38.0)	105 (40.5)	
Primary	40 (9.4)	14 (8.4)	26 (10.0)	
Secondary	84 (19.8)	31 (18.7)	53 (20.5)	
Higher	133 (31.3)	58 (34.9)	75 (29.0)	
Current employment status				0.083
Employed	128 (30.1)	58 (34.9)	70 (27.0)	
Unemployed	297 (69.9)	108 (65.1)	189 (73.0)	
BMI (kg/m^2^)				0.834
Underweight	8 (1.9)	4 (2.4)	4 (1.5)	
Normal	111 (26.1)	46 (27.7)	65 (25.1)	
Overweight	159 (37.4)	61 (36.7)	98 (37.8)	
Obese	147 (34.6)	55 (33.1)	92 (35.5)	
Family history of any disease				
Hypertension	293 (68.9)	109 (65.7)	184 (71.0)	0.242
Hyperlipidemia	124 (29.2)	48 (28.9)	76 (29.3)	0.925
Diabetes Mellitus	198 (46.6)	79 (47.6)	119 (45.9)	0.740
Renal Failure	42 (9.9)	21 (12.7)	21 (8.1)	0.126
Duration of hypertension				0.206
< 6 months	45 (10.6)	18 (10.8)	27 (10.4)	
6 months-2 years	80 (18.8)	40 (24.1)	40 (15.4)	
2.1–5 years	109 (25.6)	36 (21.7)	73 (28.2)	
5.1–15 years	132 (31.1)	49 (29.5)	83 (32.0)	
> 15 years	59 (13.9)	23 (13.9)	36 (13.9)	
Comorbidities				
Diabetes Mellitus	167 (39.3)	67 (40.4)	100 (38.6)	0.718
Chronic Kidney Disease	59 (13.9)	21 (12.7)	38 (14.7)	0.604
Coronary Artery Disease	56 (13.2)	20 (12.0)	36 (13.9)	0.582
Heart Failure	22 (5.2)	8 (4.8)	14 (5.4)	0.698
Mean systolic blood pressure (mmHg)	142.98 ± 23.052	127.38 ± 10.627	152.97 ± 23.329	.000*
Mean diastolic blood pressure (mmHg)	89.29 ± 13.658	81.87 ± 9.777	94.05 ± 13.681	.000*

A remarkable 400 (94.1%) were aware that excessive salt consumption is a risk factor for hypertension. Furthermore, 362 (85.2%) were aware that hypercholesterolemia played a significant role in the pathophysiology of hypertension. Lack of exercise, smoking, and alcohol abuse was identified as risk factors for developing hypertension by 59.5%, 48.2%, and 41.6%, respectively. In terms of practices, 324 (76.2%) had low physical activity, 395 (92.9%) were not current smokers, 421 (99.1%) were non-alcoholics, 196 (46.1%) consumed fruits 5–7 days per week, 230 (54.1%) included veget ables 1–4 days weekly in their diet, and 321 (75.5%) were habitual of using low-fat dairy products in their diet. However, none of these were significant in either the controlled or uncontrolled hypertension groups. Table 2 demonstrates the data pertaining to knowledge, attitude and practice in individuals with controlled and uncontrolled hypertension.

**Table 2:** Comparison of knowledge, attitude and treatment in patients with controlled and uncontrolled hypertension.

	Overall n (%) N = 425	Controlled hypertension n (%) N = 166	Uncontrolled hypertension n (%) N = 259	P-value
Awareness				
High dietary salt intake is a risk factor of HTN	400 (94.1)	154 (92.8)	246 (95.0)	0.345
Lack of exercise is a risk factor of HTN	253 (59.5)	95 (57.2)	158 (61.0)	0.439
Smoking is risk factor of HTN	205 (48.2)	75 (45.2)	130 (50.2)	0.313
Alcohol abuse is a risk factor of HTN	177 (41.6)	69 (41.6)	108 (41.7)	0.978
High cholesterol is a risk factor of HTN	362 (85.2)	138 (83.1)	224 (86.5)	0.342
Aware about the complications of HTN	266 (62.6)	104 (62.7)	162 (62.5)	0.983
Practices				
Physical activity				0.302
Low	324 (76.2)	132 (79.5)	192 (74.1)	
Moderate	95 (22.4)	33 (19.9)	62 (23.9)	
High	6 (1.4)	1 (0.6)	5 (1.9)	
Current smoking status				0.619
Yes	30 (7.1)	13 (7.8)	17 (6.6)	
No	395 (92.9)	153 (92.2)	242 (93.4)	
Current Alcohol status				0.281
Yes	4 (0.9)	3 (1.8)	1 (0.4)	
No	421 (99.1)	163 (98.2)	258 (99.6)	
Consumption of fruits/week				0.530
5–7 days	196 (46.1)	82 (49.4)	114 (44.0)	
1–4 days	177 (41.6)	64 (38.6)	113 (43.6)	
Never	52 (12.2)	20 (12.0)	32 (12.4)	
Consumption of vegetables/week				0.966
5–7 days	181 (42.6)	71 (42.8)	110 (42.5)	
1–4 days	230 (54.1)	90 (54.2)	140 (54.1)	
Never	14 (3.3)	5 (3.0)	9 (3.5)	
Consume low fat dairy products				0.694
Yes	321 (75.5)	124 (74.7)	197 (76.1)	
No	104 (24.5)	42 (25.3)	62 (23.9)	
Treatment				
Class of anti-hypertensive				
ACEi/ARBs	216 (50.8)	76 (45.8)	140 (54.1)	0.096
Beta blockers	130 (30.6)	47 (28.3)	83 (32.0)	0.415
Calcium channel blockers	198 (46.6)	84 (50.6)	114 (44.0)	0.184
Diuretics	34 (8.0)	11 (6.6)	23 (8.9)	0.403
Others	28 (6.6)	7 (4.2)	21 (8.1)	0.206
Number of anti-hypertensive drugs				0.422
0	11 (2.6)	4 (2.4)	7 (2.7)	
1	269 (63.3)	113 (68.1)	156 (60.2)	
2	107 (25.2)	37 (22.3)	70 (27.0)	
3 or more	38 (8.9)	12 (7.2)	26 (10.0)	

Taking into account the treatment regimen of the participants in this study, it was discovered that 216 (50.8%) were using angiotensin-converting enzyme (ACE) inhibitors, while calcium channel blockers came in second with a frequency of 198 (46.6%). Although the vast majority of 269 (63.3%) had only one anti-hypertensive drug in their management, it was shocking to see that the number of anti-hypertensive drugs had no substantial role in controlling hypertension (p = 0.422). (Table 2).

Based on the results of multivariate logistic regression analysis, age (p = 0.03) and female gender (p = 0.03) were identified as statistically significantly risk factors of uncontrolled BP (Table 3).

**Table 3:** Multivariate logistic regression to identify the risk factors associated with uncontrolled hypertension. *Statistically significant p-value (p < 0.05). Abbreviations: CI, confidence interval; Ref, Reference.

Variables	Adjusted Odds ratio (95% CI)	P-value
Age groups (years)		0.03*
20–29	Ref	
30–39	1.55 (0.34–7.14)	0.57
40–49	1.90 (0.45–8.01)	0.38
50–59	0.71 (0.17–2.95)	0.64
60 or above	1.03 (0.25–4.28)	0.97
Sex		
Male	Ref	
Female	1.80 (1.08–3.00)	0.03*
Level of education		0.90
No education	1.21 (0.64–2.32)	0.56
Primary	1.17 (0.48–2.82)	0.73
Secondary	1.27 (0.66–2.42)	0.48
Higher	Ref	
BMI (kg/m^2^)		0.88
Normal	Ref	
Underweight	1.47 (0.31–6.87)	0.63
Overweight	1.71 (0.37–8.00)	0.49
Obese	1.54 (0.33–7.23)	0.59
Comorbidities		
Diabetes Mellitus (Ref: Not reported)	0.89 (0.58–1.39)	0.61
Chronic Kidney Disease(Ref: Not reported)	0.72 (0.38–1.37)	0.62
Coronary Artery Disease (Ref: Not reported)	1.17 (0.61–2.24)	0.65
Heart Failure (Ref: Not reported)	0.92 (0.35–2.46)	0.88
Others (Ref: Not reported)	1.00 (0.50–2.00)	1.00
**Duration of hypertension**		
<6 months	Ref	
6 months-2 years	0.57 (0.25–1.29)	0.18
2.1–5 years	1.21 (0.54–2.72)	0.64
5.1–15 years	1.09 (0.49–2.41)	0.83
>15 years	1.05 (0.42–2.61)	0.92
**Awareness**		
High dietary salt intake is a risk factor of HTN (Ref: Not reported)	1.42 (0.57–3.55)	0.45
Lack of exercise is a risk factor of HTN (Ref: Not reported)	1.22 (0.74–2.02)	0.44
Smoking is risk factor of HTN (Ref: Not reported)	2.11 (0.91–4.88)	0.08
Alcohol abuse is a risk factor of HTN (Ref: Not reported)	0.54 (0.24–1.24)	0.15
High cholesterol is a risk factor of HTN (Ref: Not reported)	1.09 (0.56–2.10)	0.81
Aware about the complications of HTN (Ref: Not reported)	0.96 (0.59–1.56)	0.87
Practices		
Physical activity		0.47
Low	Ref	
Moderate	1.30 (0.75–2.24)	0.35
High	2.73 (0.28–27.0)	0.39
Current smoking status (Ref: Not reported)	1.30 (0.52–3.26)	0.57
Current Alcohol status (Ref: Not reported)	0.74 (0.05–10.3)	0.82
Consumption of fruits/week		0.33
5–7 days	0.72 (0.35–1.47)	0.37
1–4 days	1.03 (0.50–2.10)	0.94
Never	Ref	
Consumption of vegetables/week		0.94
5–7 days	0.90 (0.26–3.17)	0.87
1–4 days	0.85 (0.24–2.97)	0.79
Never	Ref	
Consume low fat dairy products (Ref: Not reported)	0.90 (0.54–1.50)	0.68
Number of anti-hypertensive drugs		0.57
0	Ref	
1	1.10 (0.28–4.30)	0.90
2	1.47 (0.36–6.03)	0.59
3 or more	1.67 (0.36–7.82)	0.52

## 4 Discussion

The health system is endeavoring to combat the burden of infectious diseases that have subjugated the subcontinent. During this time of struggle, the rising trends in hypertension and its consequences are further creating an exigent situation for the healthcare departments. This study assisted us in evaluating the degree of awareness and adherence to healthy lifestyle practices amongst hypertensive participants. Healthy lifestyle behavior plays an important role not only in the control of hypertension but also in minimizing its complications. In addition, this study elaborates the influence of multiple risk factors and demographics along with the knowledge, attitude, and treatment on the patients with controlled as well as uncontrolled hypertension.

According to our study, the prevalence of hypertension was 65.6% in women as compared to 34.4% in males, therefore showing a significant difference in both genders. This is continuous with the findings of Mirzae. M et al. [19] and Tabrizi. JS et al. [20] while contradictory to the findings of Egbhali. M et al. [21]. Higher prevalence in females is possibly due to increased BMI secondary to physical inactivity and sedentary lifestyle. Usage of oral contraceptive pills, vitamin D deficiency, and menopause in females may also contribute as a notable risk factor in the development of hypertension [20].

As per the results of this study, the most common age group affected with hypertension was between 50 and 59 with a prevalence of 33.9% corroborating with the study of Penpid. S et al. [22] and Sanuade. OA et al. [23] which revealed an approximately similar value. Overall, the incidence is increasing with age similar to a study conducted by Maharjan. B [24] showing a much higher rate of 59.1% among the age group of 50–59. In old age, standard anatomy and physiology of the arterioles is altered resulting in increased stiffness and decreased elasticity of the vessels. This disrupts the mechanism of arteriolar resistance with sudden hemodynamic changes constituting as the likely rationale of increased blood pressure in advancing age [25]. Both, age and gender had a similar trend in controlled in uncontrolled groups, thereby depicting similar pathophysiology in each of these groups respectively.

Literature reveals a strong association between levels of education with hypertension [26,27]. Better perception and knowledge can benefit an individual to improve their blood pressure. However, our results did not exhibit an extremely significant correlation between the two. Even though the prevalence of uncontrolled hypertension was 40.5% in people without education and 29.0% in people with higher education as compared to 38.0% and 34.9% in the controlled group respectively, overall the difference was not noteworthy. This was similar to what Katibeh. M et al. stated in their study [28]. The health literacy of an individual is far more important than the knowledge about health care and knowledge regarding this particular disease will yield a better outcome [19].

BMI plays a vital role in the evolution of hypertension. Obesity is a strong predisposing factor towards increased blood pressure and occurs secondary to lower physical activity and consumption of unhealthy food with less nutrition and more cholesterol [29,30]. Our results explicit a mild difference between the controlled and uncontrolled HTN among obese and normal-weight individuals. People with normal and obese weight had a prevalence of 27.7% and 31.1% in the controlled group while 25.1% and 35.5% respectively in the uncontrolled one. This elucidates that other factors such as genetic predisposition along with diverse environmental stimulants may lead to hypertension in the absence of obesity [31]. It is also supported by a finding in our study which shows that there is a higher prevalence of hypertension in individuals with a past family history of this disease.

This study exhibits that majority of the hypertensive patients were well aware of certain risk factors such as salt reduction, physical inactivity, high cholesterol, alcohol, and smoking. However, no significant difference was observed between the levels in controlled and uncontrolled groups. This is possibly due to lack of applied knowledge, dissent towards the treatment strategies, recalcitrant towards lifestyle modifications, and other preventive measures. Moreover, deficiency of proper health care services and untrained management staff may also contribute to this result.

Moreover, the population is aware of the health effects of smoking but many of them fail to identify its association with raised BP. This was supported by the result that only 48.7% were cognizant of the fact that smoking can increase the risk of hypertension. Results were consistent with Tesema et al. (56.9%) [32], but a greater percentage was reported by Bilal et al., in 2015 [7]. This increase in awareness of smoking hazards is a good indication but further advances should be made to break the chain of smoking especially in youth. However, our study could not prove this relationship between smoking and hypertension in controlled and uncontrolled groups. Since nonsmokers were the predominant participant in our study, thereby providing considerable justification for our result. It is further supported by the synchronous finding in a study conducted by Pengpid S^22^ showing no correlation between the two. This may be due to retarded effect of nicotine, or by tolerance and adaptation towards nicotine thereby negating it as a contributing factor towards hypertension. Different studies have generated different outcomes regarding this correlation therefore, it remains a conflict so far [33].

Our study revealed no valuable difference between physical activity and hypertension among the control and uncontrolled group. This finding corresponds with the result compiled by Katibeh et al. revealing no remarkable correlation [28]. Physical exercise effectuates vascular resistance and ultimately leads to vasodilation by altering the renin-angiotensin-aldosterone system, thereby reducing the risk of hypertension. However, this association was not significant in our study and requires further investigation and specification regarding the time duration, regularity, and type of exercise. Only a few alcoholics were recognized in the current research and they were far less than stated by Neupane et al. [34]. It's most likely due to religious restrictions and cultural obligations rather than hypertension monitoring.

This study did not reveal a substantial outcome of fruits, vegetables, low-fat products among both groups. This may be due to a better lifestyle modification by the patients diagnosed with hypertension and the data collected after the correction of dietary habits. Moreover, a healthy diet such as fruits and vegetables may improve the illness, but other elements such as non-compliance towards pharmacological management along with the refusal to remaining lifestyle modification may be the dominating component towards this result. Nevertheless, the adoption of the DASH diet (comprising of plenty of fruits and vegetables, low-fat dairy products, and vegetable oils) in adjunct to pharmacological therapy is an effective way of managing hypertension and its complications [35].

Treatment provided was also similar between both the groups, again showing no considerable difference between the outcomes. The ACE inhibitor was the first-line drug used in many followed by calcium channel blocker. Our results generated poor control of this disease, regardless of the number of medications adjoined in the existing therapy. This is likely due to negligence and non-adherence towards the regimen and frequent changes of medications [20].

## 5 Limitations

This study was carried out in a single center; further multicenter studies are required to strengthen these shreds of evidence for the lifestyle of hypertension. In this study we enrolled only hospital-based hypertensive participants; a community-based survey of hypertensive individuals will provide more significant results. Since it was a cross-sectional study, therefore, cannot predict the casualty rate and a proper association of hypertension with independent variables. Apart from the blood pressure readings, the entire results were derived based on information provided by the patient. There may be biasness on account of self-reported details. Moreover, detailed information about the adherence of the drug, economic status, and healthcare facilities was not taken into account.

## 6 Conclusion

There was no significant difference in the statistics of controlled and uncontrolled hypertensive patients. This requires further investigation and evaluation to identify the unknown risk factors and co-morbid contributing to these findings. Most of the patients are on treatment and still not controlled, and thus could be considered under treatment. Interventions should be considered and implemented to potentially increase the control rates. One example is to consider initiating two anti-hypertensive agents upon the diagnosis of hypertension and then to up-titrate dosage and add a third and fourth agent in short follow-up intervals until blood pressure control was achieved. Furthermore, health professional's advice and counseling skills, social media, internet, and public awareness sessions can play an active role in the management of BP and its associated complications.

## Ethical approval

This study received ethical approval from Jinnah Medical and Dental College.

## Sources of funding

None.

## Author contribution

All authors made substantial contribution to this article.

## Registration of research studies

1. Name of the registry:
2. Unique Identifying number or registration ID:
3. Hyperlink to your specific registration (must be publicly accessible and will be checked):

## Guarantor

N/A.

## Consent

Informed verbal consent was obtained from patients.

## Provenance and peer review

Not commissioned, externally peer-reviewed.

## Declaration of competing interest

None declared.
