# Supplementary figures and images for: Awareness, treatment, and practices of lifestyle modifications amongst diagnosed hypertensive patients attending the tertiary care hospital of Karachi: A cross-sectional study
Source: Ann Med Surg (Lond). 2022 Sep 13;82:104587. doi: 10.1016/j.amsu.2022.104587 (PMC9577521; doi:10.1016/j.amsu.2022.104587)

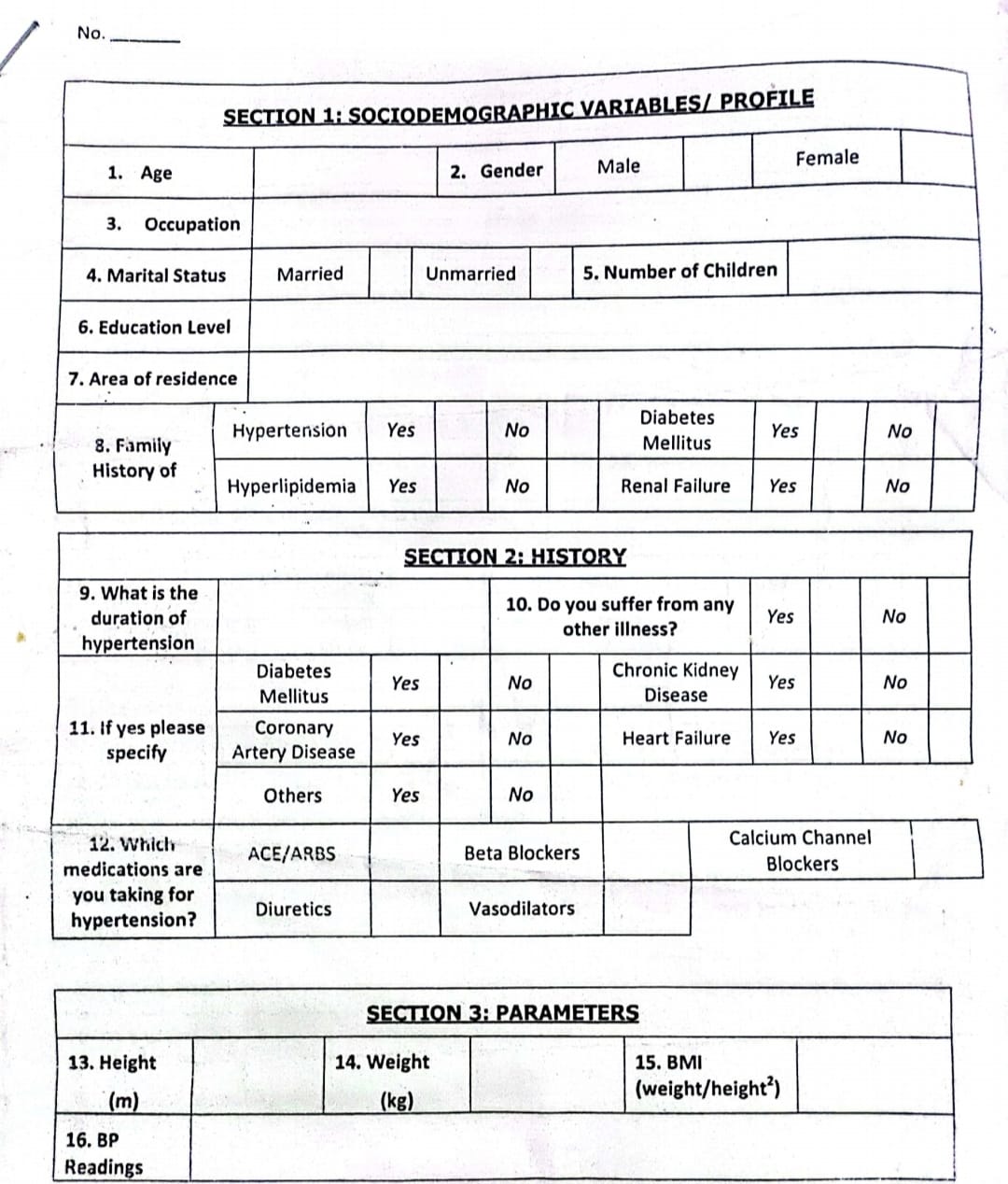


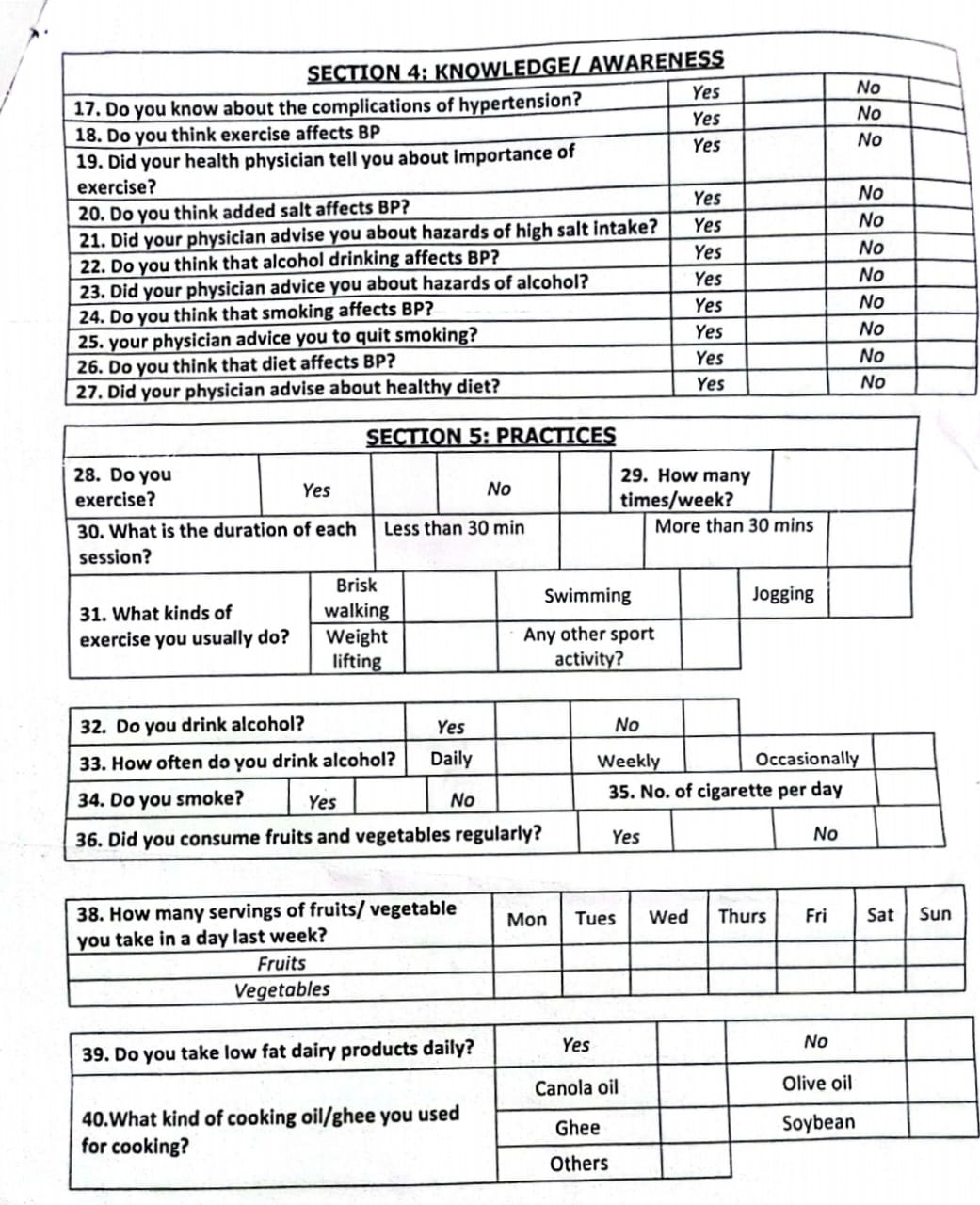

Supplement: Multimedia component 2 [file mmc2.docx]
